# Obesity or BMI Paradox? Beneath the Tip of the Iceberg

**DOI:** 10.3389/fnut.2020.00053

**Published:** 2020-05-07

**Authors:** Lorenzo Maria Donini, Alessandro Pinto, Anna Maria Giusti, Andrea Lenzi, Eleonora Poggiogalle

**Affiliations:** Department of Experimental Medicine, Sapienza University, Rome, Italy

**Keywords:** obesity, obesity paradox, nutritional status, body composition, body mass index

## Abstract

The obesity paradox refers to extant evidence showing that obesity in older subjects or in patients with several chronic diseases may be protective and associated with decreased mortality. A number of mechanisms have been postulated to support the existence of obesity paradox; however, marked heterogeneity was found across studies and this has cast doubt on the actual presence of this phenomenon. The aim of the present narrative review is to summarize evidence underlying the concept of obesity paradox, focusing on limitations and bias related to this phenomenon, with emphasis on the use of body mass index (BMI). A major cause of the discrepancy between studies may be related to the use of BMI in the definition of obesity, that should consider, instead, excess body fat as the main characteristic of this disease and as the unique determinant of its complications. In addition, the adjustment for potential confounders (e.g., stage and grade of diseases, smoking habit, inability to capture the presence of signs of undernutrition in the normal-weight comparative group, consideration of body composition) may significantly scale down the protective role of obesity in terms of mortality. However, it is still necessary to acknowledge few biases (e.g., reverse causation, attrition bias, selection bias of healthy obese subjects or resilient survivors) that would still apply to obesity even when defined according with body composition. Further research should be prompted in order to promote correct phenotyping of patients in order to capture properly the trajectories of mortality in a number of diseases.

## Introduction

The obesity paradox refers to extant evidence showing that obesity in older subjects or in patients with several chronic diseases may be protective and associated with decreased mortality. Gruberg et al. (1) first observed that overall mortality (1 year follow-up) was significantly higher in patients with coronary artery disease after percutaneous coronary intervention and normal body mass index (BMI) compared to overweight/obese subjects. Since then, a number of studies, encompassed by the umbrella term “reverse epidemiology,” found that obesity, hypercholesterolemia, and hypertension were associated with improved survival among dialysis patients (2), in chronic heart failure (CHF) (3), after acute myocardial infarction (4), in chronic obstructive pulmonary disease (5), in older nursing home residents (6), in peripheral arterial disease, in stroke and thromboembolism, in post-operative complications during catheter ablation for atrial fibrillation and after cardiac surgery, in surgical intensive care unit, in patients undergoing non-bariatric surgery, in type 2 diabetes (reducing amputation risk among non-elderly diabetic men), and in critically ill and osteoporosis patients (7).

The aim of the present narrative review is to summarize evidence underlying the concept of obesity paradox, focusing on limitations and bias related to this phenomenon, with emphasis on the use of BMI.

## Biological Hypotheses and Mechanisms Underlying the Obesity Paradox

Different mechanisms have been postulated to support the existence of obesity paradox.

Body structure and body composition: increased body weight may hinder the metabolic consequences of diseases and of treatments by providing adequate muscle and adipose reserves (8).

Lipid metabolism: high levels of total cholesterol and lipoproteins may improve the endotoxin-scavenging effect, while patients with CHF and low total serum cholesterol level are more prone to endotoxemia and its inflammatory consequences due to bacterial/endotoxin translocation from bowel wall edema (3).

The release of N-terminal pro-B-type natriuretic peptide (NT-proBNP) by cardiomyocytes, due to increased wall tension, may be considered as a major prognostic factor for mortality in acute coronary disease. NT-proBNP levels are significantly reduced in patients who are overweight or obese compared to subjects with a lower BMI after myocardial infarction (4).

Prothrombotic factors (e.g., thromboxane B2) are negatively correlated with BMI and leptin since their production is influenced by endothelial function that is paradoxically better in subjects with obesity than in non-obese individuals (4).

Increase in ghrelin production/sensitivity has been showed to be a compensatory mechanism to hinder the evolution of heart failure, since it may improve cardiac contractility by increasing left ventricular function and exercise capacity, while it reduces muscle wasting in patients with CHF; ghrelin also affects appetite and can be responsible for a parallel rise in food intake and weight gain (9).

Cytokines production: cardiometabolic risk is associated with augmented production of cytokines (e.g., tumor necrosis factor TNF-α). The production, by subcutaneous adipose tissue, of soluble TNF-α receptors I and II, which is correlated with BMI and percent body fat, in patients with heart failure, is lower in subjects with obesity. These receptors are supposed to bind TNF-α and to counteract its negative effects on the myocardium (7). Several adipokines (e.g., adiponectin, apelin, omentin, and others) produced by adipose tissue have shown to be cardioprotective and to exert a variety of favorable effects on cardiovascular function (10).

Endothelial/vascular aspects: increased mobilization of endothelial progenitor cells may protect patients with severe obesity from atherogenesis through promotion of regeneration processes in the damaged myocardium and the development of new blood vessels. This process leads to the reduction of the afterload due to higher flow-mediated dilation and lower intima-media thickness, to the enhancement of myocardial contractile function and metabolic processes in cardiomyocytes, to the reduction of apoptosis and fibrosis of the myocardium (11).

Cancer biology: obesity seems to be associated with lower stage disease, smaller tumor size, and less aggressive biological subtypes. Moreover, overweight and obesity may positively influence treatment outcome since excess adipose tissue affects pharmacokinetics of cancer treatment regimens, while providing a nutritional supply to deal with surgical and anticancer treatments (12, 13).

## Limitations to the Studies Assessing the Presence of the Obesity Paradox

Significant heterogeneity was found across studies supporting the presence of the obesity paradox (e.g., study population, degree of control for confounding factors, length of follow-up), and this has cast doubt on the actual existence of this phenomenon (14, 15).

A wide range of normal BMIs (18.5–25.0 kg/m^2^) may include heterogeneous groups, and mortality rates tend to be significantly higher at the lower end of the BMI range (15). In a systematic review conducted by Flegal et al. (16) (97 studies, around 3 million individuals, more than 270,000 deaths), the “obesity paradox” was significantly downsized. All-cause mortality was significantly greater in patients with BMI ≥ 35 kg/m^2^ compared to normal weight subjects. Class I obesity (BMI 30- 34.9 kg/m^2^) was not associated with greater all-cause mortality, and overweight was associated with a significantly lower mortality rate. Moreover, none of the different classes of BMI was associated with mortality in subjects aged 65 years and older. Another systematic review concerning obesity in the elderly (17) confirmed that obesity represents a mortality risk in older adults, but with different BMI thresholds compared to adult population (18). A U-shaped-curve correlation between BMI and mortality has been shown with an increased risk of death for low (<18.5 kg/m^2^) as well as very high BMI values (> 35 kg/m^2^). But the nadir of the curves differs from what is known in younger obese subjects, and we can hypothesize a shift of the nadir toward a higher BMI (between 23.5 and 27.5 kg/m^2^) in the elderly, which is at least 1–5 points higher than that in young and middle-aged adults (17).

Also, a selection bias has been accounted for by different authors (3, 4, 19, 20). Patients with obesity often present with comorbidities, and they undergo medical check-ups more frequently and consequently, all diseases associated with obesity paradox may have been diagnosed at earlier stages (12). Subjects with obesity, especially those affected by high levels of comorbidity, are more prone to early death and cannot be included in later cohorts. Thus, the obese population represented in these studies is characterized by obese but likely healthier individuals (15).

The increased survival of patients with high BMI may also be related to the lack of consideration, in the cohorts with lower BMI, though within the normal BMI range, of subjects with extremely low BMI and of causes explaining low BMI: significant unintentional weight loss due to the presence of high levels of comorbidity (e.g., greater predisposition to develop bleeding and anemia; higher prevalence and severity of hypertension and valvular regurgitation, chronic obstructive pulmonary disease, arrhythmias, infectious diseases) and of the “malnutrition-inflammation complex syndrome” (MICS): in coronary heart disease and dialysis patients, both protein-energy malnutrition and inflammation, or the combination of the two, are more frequent compared to the general population, while different aspects of MICS (e.g., low weight-for-height, hypocholesterolemia, hypocreatininemia) may be considered as risk factors of poor outcome in CHF and dialysis patients (20, 21). Comparing subjects with overweight or obesity to subjects belonging to this heterogeneous stratum may lead to a misinterpretation of the correlation between BMI and mortality (22).

In addition, in some studies, the protective effect of obesity was found in subjects who were significantly younger than their normal-weight counterparts (younger subjects usually have less severe coronary heart disease, a preserved cardiac function and thus better survival rates) (23) or in elders with overweight or obesity who could be considered “resistant” to negative consequences of higher BMI at younger age (6). On the other hand, subjects who were normal weight at the time of death could represent a high-risk group for mortality because of unintentional weight loss due to hormonal changes, decreased appetite, and/or chronic undetected medical or mental illness (22). Finally, some normal-weight subjects may have previously been obese but have lost weight due to illness (reverse causality), hence representing a high-risk of mortality with normal or low BMI being the consequence of a significant illness (13).

Several studies accounting for the presence of an obesity paradox have an important performance bias, since more appropriate medical treatments were administered to patients with a high BMI than in those with a normal BMI (4, 6, 7), together with an attrition bias (3, 6). The median follow-up period of these studies (around 2 years) could have been too short to show negative effects of obesity, while undernutrition may have a greater impact on mortality in a reduced period of time (time discrepancy). In a study conducted by Nigam et al. (24), comparing three different classes of BMI (<25; 25–29.9; ≥ 30 kg/m^2^), different mortality risks were described for subjects with overweight or obesity in the short term (<6 months) compared to a longer period of observation. In addition, among the three classes of BMI, they observed that incidence of cardiac-related mortality in the long term was higher in subjects with overweight or obesity than that observed in the normal BMI population. After myocardial infarction, the reduced obesity survival paradox was explained by younger age at the time of initial infarction and by a reduced prevalence of non-cardiovascular comorbidities (24).

Timing of BMI ascertainment may also significantly influence obesity paradox: different studies considered BMI assessed several years before, whereas other studies, which used BMI calculated at diagnosis or several months to 1–2 years after cancer diagnosis, did not find any association or lower mortality with higher BMI.

Similarly, timing of diagnosis of diseases, such as cardiovascular disease, in patients with obesity may occur earlier than in normal-weight subjects because presence of the obesity, and this can be at the origin of a lead time bias (25).

Other studies did not control for race/ethnicity or sex, while obesity-mortality association seems to be affected by these variables and obesity paradox is more evident in men than in women (26).

Finally, confounding factors have not been always considered in those studies. In fact, the adjustment for potential confounders may scale down the protective association of obesity with mortality (27–30). In a study conducted by Hakimi et al. (31) the association of higher BMI with reduced cancer-specific mortality was lost after adjusting for cancer stage and grade. Confounding by smoking is another major threat to BMI-mortality analysis. Indeed, differences in intensity, inhalation, frequency, and duration of smoking habit, and its association with lower body weight, may represent an important limitation to studies concerning obesity paradox (13, 22).

## The Case of BMI as a Proxy of Obesity

Although observed associations between obesity and mortality do not prove causality, a major cause of the discrepancy across studies may be related to the use of BMI in the definition of obesity that should consider, instead, excess body fat as the main characteristic of this disease and as the unique determinant of its complications (32). BMI represents the sum of fat-mass index (FMI) and fat-free mass index (FFMI) (33). The latter accounts for skeletal muscle mass, bone, and organs, while FMI is composed of peripheral and visceral adipose tissues. All these components of BMI have different roles in contributing to health status, and changes in BMI are not related to a proportional and linear modification of body compartments (34). For these reasons, different authors have pointed out the limitations of BMI in defining nutritional status (35–37): BMI fails to reflect adiposity and body composition (and their distribution), and to detect “normal weight obese” subjects (38), patients with sarcopenic obesity (39), and the presence of undernutrition in overweight subjects (40, 41); BMI varies depending on sex (men and women do not have the same body composition at similar levels of BMI) and ethnicity (Asians, Chinese, and Aboriginal people have similar metabolic risk factors at significantly lower—~6 kg/m^2^–BMI values compared to Caucasians) (42); BMI fails to account for fitness related to the proportion of lean mass to adiposity (43). Fat-free mass (FFM) is strictly correlated to cardiorespiratory fitness and to physical functional abilities (43). The correlation between BMI and mortality tends to be modified by the cardiorespiratory fitness status, as it happens in chronic-obstructive pulmonary disease (COPD): the risk of death in unfit men is two-fold higher compared to fit men regardless of obesity status (44). Caan et al. (45) have shown that body composition may partially explain the U-shaped association between BMI and cancer (e.g., colorectal cancer) survival. The correlation between BMI and fat mass (FM)—especially in subjects with obesity—is not linear, while the relationship between BMI and FFM tends to be linear. Therefore, higher BMI values are frequently associated with higher FFM (and not necessarily to obesity or to an increase of FM) and cancer patients who are overweight or obese have higher levels of lean mass than their normal-weight counterparts. On the contrary, lower BMI (and lower lean mass) is associated with higher risk of recurrence, surgical complications, treatment-related toxicities, and overall and cancer-specific mortality (45–48).

Similar results were found by Lin et al. (49) in patients with chronic kidney disease. Using BMI cut-points, 27.9% of patients were obese; while agreeing with the definition based on body fat percentage, the prevalence of obesity raised to 48.8% with a marked percentage of patients (29.4%) who had excess body fat with a normal BMI. When adjusting the regression models for either BMI or body fat percentage, obesity defined by BMI was associated with a significantly lower mortality hazard ratio (HR: 0.23; 95% CI: 0.07–0.71; *p* = 0.011), whereas the result was inverted when obesity was defined by body fat percentage (HR: 2.75; 95% CI: 1.28–5.89; *p* = 0.009). Subjects with excess fat mass, irrespective of BMI, were characterized by a reduced lean mass (e.g., sarcopenic obesity) and had higher death risk compared with patients with obesity defined by both BMI and body fat (HR: 5.11; 95% CI: 1.43–18.26; *p* = 0.012) (49).

Nonetheless, regardless of body composition, conflicting results emerged when using markers of central obesity in place of BMI. In a systematic review by Coutinho et al. (50), BMI, waist circumference, and waist-to-hip ratio were compared against mortality outcome in coronary artery disease (CAD) patients. Interestingly, central obesity was positively associated with higher mortality in individuals with CAD, whereas BMI was inversely associated with mortality. The effect of central obesity on mortality was observed even in patients with normal BMI (50).

Use of body composition analysis is indeed an attempt to overcome the misleading properties of BMI: in an elegant study by Gonzalez et al. (48), obesity paradox was explored in cancer patients using either BMI or body composition obtained by bioimpedance analysis, indicating that obesity paradox emerged when using BMI, but it was not confirmed by analyses based on body composition. Though just a minority of studies investigating the obesity paradox relied on body composition assessment, evidence supports the role of low lean mass as the actual predictor of mortality when used in place of BMI (51).

## Conclusion

The actual paradox seems to be keeping defining obesity using BMI, which is not able to quantify body fat percentage and adiposity distribution, nor the degree of metabolic disturbances that it can underlie. In fact, obesity is characterized by a significant complexity related to alterations of nutritional status (energy and nutrient intake, body composition), to the interaction of psychological and social factors, to functional impairment, to hormonal and metabolic alterations, to the impairment of different organs (e.g., cardiovascular and respiratory systems) and quality of life that cannot be adequately described by BMI.

However, replacing BMI by body composition is not an easy fix for the issue of the obesity paradox: some of the above mentioned biases reported in previous studies would still apply to obesity even when defined by body composition methods, such as reverse causation and selection bias of healthy obese subjects or resilient survivors. In addition, no universal cut-points have been yet defined to classify obesity based on body fat that are accurate by sex, ethnicity, age, or physiological groups (e.g., post-menopausal women).

Body composition phenotypes, taking into account both body fat and lean mass, and metabolic and functional variables, and duration of obesity (as well as of normal weight), can capture properly the trajectories of mortality in a wealth of diseases. Further research should be prompted in order to promote correct phenotyping of patients. The obesity paradox is just a lesson to be learned.

## Author Contributions

LD led the study design, was actively involved in the study conception, design, strategic decisions, and drafted the manuscript. AL, AP, AG, and EP contributed to the analysis of the literature, interpreted the findings, and helped in drafting the manuscript. AL and EP participated in the study design and coordination and gave intellectual inputs on the manuscript. All authors have read and approved the manuscript.

## Conflict of Interest

The authors declare that the research was conducted in the absence of any commercial or financial relationships that could be construed as a potential conflict of interest.
